# Recommendations for using analytical anisotropic algorithm and AcurosXB for epidermal dose calculations in breast radiotherapy from an in vivo Gafchromic film study

**DOI:** 10.1002/acm2.14416

**Published:** 2024-05-29

**Authors:** Aria Malhotra, Emilie E. Carpentier, Cheryl Duzenli

**Affiliations:** ^1^ BC Cancer Vancouver British Columbia Canada; ^2^ Department of Physics and Astronomy University of British Columbia Vancouver British Columbia Canada; ^3^ Department of Surgery Division of Radiation Oncology and Experimental Radiotherapeutics University of British Columbia Vancouver British Columbia Canada

**Keywords:** breast radiotherapy, Eclipse AAA and AXB algorithms, GafChromic film, in vivo skin dose

## Abstract

**Background and purpose:**

This study recommends clinical epidermal dose calculation methods based on in‐vivo film measurements and registered skin dose distributions with the Eclipse (Varian Medical Systems) treatment planning system's Analytical Anisotropic Algorithm (AAA) and Acuros XB (AXB) dose calculation algorithms.

**Materials and methods:**

Eighteen AAA V13.6 breast plans were recalculated using AXB (dose to medium) V13.5 with the same beam parameters and monitor units as in the original plans. These are compared against in‐vivo Gafchromic film measurements from the lateral and inferior breast regions. Three skin structures in the treatment planning system are evaluated: a surface layer of voxels of the body contour, a 0.2 cm internal skin rind, and a 0.5 cm internal skin rind.

**Results:**

Systematic shifts are demonstrated between the film measurements of skin dose and the Eclipse dose calculations. On average, the dose to the surface layer of pixels is underestimated by AAA by 8% and overestimated by AXB by 3%. A 5 mm skin rind extended into the body can increase epidermal dose calculations on average by 8% for AAA and 4% for AXB.

**Conclusion:**

This is the first study to register in‐vivo skin dose distributions in the breast to the treatment planning system for comparison. Based on the results from this study it is recommended that epidermal dose is calculated with a 0.5 cm skin rind for the AAA algorithm and with rind thickness up to 0.2 cm for the AXB algorithm.

## 1 INTRODUCTION

The goal of breast radiation therapy is to deliver enough dose to effectively treat the cancerous region while minimizing dose to healthy tissue. Linear accelerator (linac) beam and field parameters must be adjusted and optimized to plan an effective treatment. Dose calculation algorithms are a fundamental tool used in treatment planning to calculate the dose being delivered to the patient. Accurate calculations are key to positive clinical outcomes. Within the Eclipse (Varian Medical Systems, Palo Alto, California, USA) treatment planning system (TPS), there are two primary external beam photon algorithms: the Analytical Anisotropic Algorithm (AAA) and AcurosXB (AXB).

AAA is a convolution and superposition dose calculation algorithm based on work by Dr. Ulmer and Dr. Kaissl.^1^, ^2^, ^3^ AXB conducts an open form solution of the linear Boltzmann transport equation (LBTE) directly as described by Vassiliev et al.^4^ AXB has been shown to be more accurate across tissue heterogeneities than AAA.^5^, ^6^, ^7^, ^8^ One instance of a heterogeneous interface is between the skin surface and air.

The most acutely responding part of the skin is the basal cell layer, which is the deepest layer within the epidermis.^9^ This layer houses the stem cells that replenish the upper layers of skin cells and radiation damage to this region can cause acute dermatitis. It is recommended to define the basal layer at a depth of 0.007 cm.^10^ The finest resolution of the Eclipse™ dose calculation algorithms is a 0.1 cm grid. The lateral breast is the entrance and exit surface of a tangential beam pair, where there is longitudinal electronic disequilibrium as the dose builds up. The inferior breast is a lateral exit surface, with the skin dose resulting from scatter within the breast volume. At this surface there is lateral electronic disequilibrium. Dose calculation algorithms might calculate the dose to these surfaces differently due to geometry. The steep dose increase, small scale of the region of interest at the interface of air and tissue, and various incident photon angles in a tangential breast treatment make epidermal dose calculation in TPS a challenge.

Epidermal dose is directly associated with skin toxicity.^9^, ^11^, ^12^ A better understanding of in‐vivo skin dose measurements and calculations in radiotherapy treatment planning could improve the planning process and help minimize the incidence of skin toxicity for patients at high risk.

## 2 MATERIALS AND METHODS

### 2.1 Study design

A research ethics board approved pilot study (ClinicalTrials.gov NCT04543851) was conducted to assess the use of a novel breast support device prototype, showing promising results for alleviating skin folds in the breast and reducing the treatment volume and dose to organs at risk.^13^ The device is composed of a carbon fiber material system with a water equivalent thickness of 0.1 g/cm^2^ and is shaped to cradle the breast, separating it from the chest surface. The breast cradle was positioned at a 12° angle with respect to the vertical, a feature designed to complement the 12° angle of the slant board on which the patients are positioned, as previously illustrated.^13^

Twenty patients with stage I to III invasive breast cancer were recruited across two centers in British Columbia, Canada between May 2018 and September 2019. Treatment planning dose was calculated using the Varian Eclipse AAA algorithm version 13.6 with a prescription of 42.5 Gy in 16 fractions or 50 Gy in 25 fractions. Out of the 20 patients in this study, 19 were treated with a tangential field‐in‐field technique, and 1 patient was treated with an enhanced dynamic wedge. Nine treatment plans used 6 MV, two treatment plans used 10 MV, eight treatment plans used a combination of 6 and 10 MV, and one treatment plan used a combination of 6 and 15 MV. In the current study, treatment plans for the 18 patients treated with 6 and 10 MV beam energies with the tangential field in field technique have been retrospectively analyzed.

### 2.2 Film measurements

**FIGURE 1:**
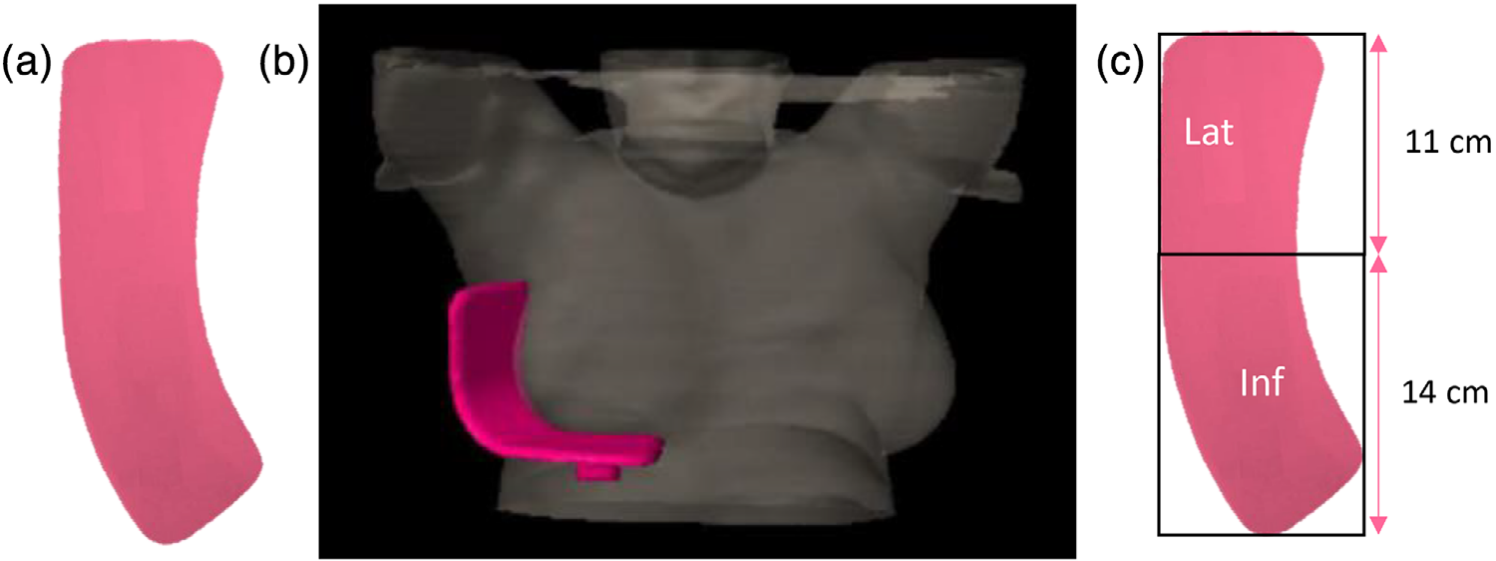
(a) Film cut from stencil to fit surface of breast cradle, flattened into 2D. (b) Breast cradle (pink contour) on a 3D reconstructed CT scan of a patient in the study. (c) Region of interest divided into lateral (lat) and inferior (inf) segments.

In vivo skin dose was measured during treatment as a surrogate for epidermal dose using Gafchromic EBT3 film (Ashland, New Jersey, USA). EBT3 film has a 2.8 × 10^−3 ^cm thick sensitive layer sandwiched between two 1.25 × 10^−2 ^cm thick polyester layers.^14^ As described in a previous study,^15^ the films were cut using a stencil to create a reproducible 170 cm^2^ region to place on the breast cradle against the skin in the lateral and inferior portion of the breast (see Figure 1a). Film measurements under the device are at an effective depth of 0.1 g/cm^2^, which is highly relevant and is consistent with the resolution of the TPS. Films were scanned using an Epson Expression 10000XL (Epson America Inc., Los Alamitos, California, USA) scanner with 72 DPI resolution at least 48 h post irradiation to ensure stable film darkening,^16^ using the one scan protocol.^17^ Dose was measured on three treatment fractions for each patient. The three film measurements were rigidly registered using automated MATLAB (R2020b; The MathWorks, Inc., Natick, Massachusetts, USA) inbuilt functions and averaged to create a mean measured dose distribution for each patient. Green channel film dosimetry produced the most accurate results in preliminary performance testing and was used in this analysis with Film QA Pro software (Ashland Inc., Bridgewater, New Jersey, USA). The two outer pixels of the film dose map were removed to eliminate any potential film cutting artefacts. A 5 × 5 pixel 2D digital Weiner filter and median filter were applied to all film data during analysis.^14^

### 2.3 AAA and AXB dose calculations

Treatment plans were calculated based on clinical standard of care CT scans with resolution 0.12 cm x 0.12 cm x 0.25 cm. Post treatment, the plans were re‐calculated for comparison with the AXB algorithm (dose to medium) version 13.5. Monitor units and beam parameters from the original plans were kept consistent in the recalculation. Accounting for computing memory issues due to field size, the smallest calculation grid size was chosen for each case. Out of the 18 patients, 15 plans had calculation grid size 0.1 cm, and 3 had calculation grid size 0.15 cm. Inaccuracy can occur at the beam's entrance point for model‐based calculations, and extending the body contour outwards into air is a technique that can help to address this.^18^ A 2.0 cm extended body contour was used to assist the TPS to improve dosimetric accuracy at the surface. This technique was implemented in this study for both AAA and AXB calculations for consistency.

The body structures were defined in the TPS with Hounsfield Unit thresholding of −1000 to −3071. The breast cradle structures were defined similarly with thresholding −800 to −600. An example is illustrated in Figure 1b. The region of the lateral and inferior breast in contact with the cradle (and thus, the film) is contoured as a structure within the TPS by creating a Boolean AND structure between the body and breast cradle contours. This structure will henceforth be referred to as the surface voxel contour. The dose in this single layer of voxels is extracted from Eclipse. Using in‐house MATLAB code, this 3D dose surface is iteratively transformed into a 2D surface structure for comparison against the film. Using the known cradle shape and calculating sequential point to point vectors along the 3D surface, points are relocated on the 2D surface, preserving the vector magnitude and angle in the 2D plane. A linear interpolation is performed on the sparser Eclipse data to match the resolution of the film. The calculated and measured dose maps are rigidly registered for accurate comparison using the MATLAB inbuilt functions previously used to register the three film measurements for each patient.

### 2.4 Film versus TPS analysis

Treatment planning dose volume histograms (DVHs) of the surface voxel contours for each patient are compared against skin rinds extending 0.2  and 0.5 cm interiorly from the surface voxel contour to compare against skin contours seen clinically and in research.^18^, ^19^, ^20^ The DVHs of the 3D surface voxel contour, the 0.2 cm rind contour, and the 0.5 cm rind contour from Eclipse are compared.

The region of interest on the breast is segmented into lateral (upper 11 cm) and inferior (lower 14 cm) sections as shown in Figure 1c. The analysis in this study is conducted on the full region of interest, the lateral surface, and the inferior surface.

The surface voxel contour is a single layer and can be flattened into 2D pixels once the structure has been unfolded for comparison. The film is masked to display only the region of contact between the skin and film based on the TPS surface voxel contour. These TPS surface pixels are compared with the 2D film dose. The calculated (AAA and AXB) and measured (film) dose distributions are described as a percent of the prescribed dose. These dose distributions are compared for each patient as a pixel‐by‐pixel difference between calculated and measured dose. These results are amalgamated into histograms showing the distribution of dose across all patients for AAA versus film and AXB versus film. A Gaussian distribution is fit to the data. A *t*‐test is conducted to determine statistically significant differences between the AAA versus film and AXB versus film distributions.

A Gamma Index Analysis (γ) is conducted as a quality assurance check on the registration of the 2D films and the unfolded TPS dose distributions. The distance to agreement set to 0.5 cm with dose agreement tolerances of 7% (γ_7%,0.5 cm_), and 10% (γ_10%,0.5 cm_). The distance to agreement of 0.5 cm was chosen due to artefacts in the dose distributions, and the dose tolerances were chosen to capture the variance in the dose difference analysis.

## 3 RESULTS

**FIGURE 2:**
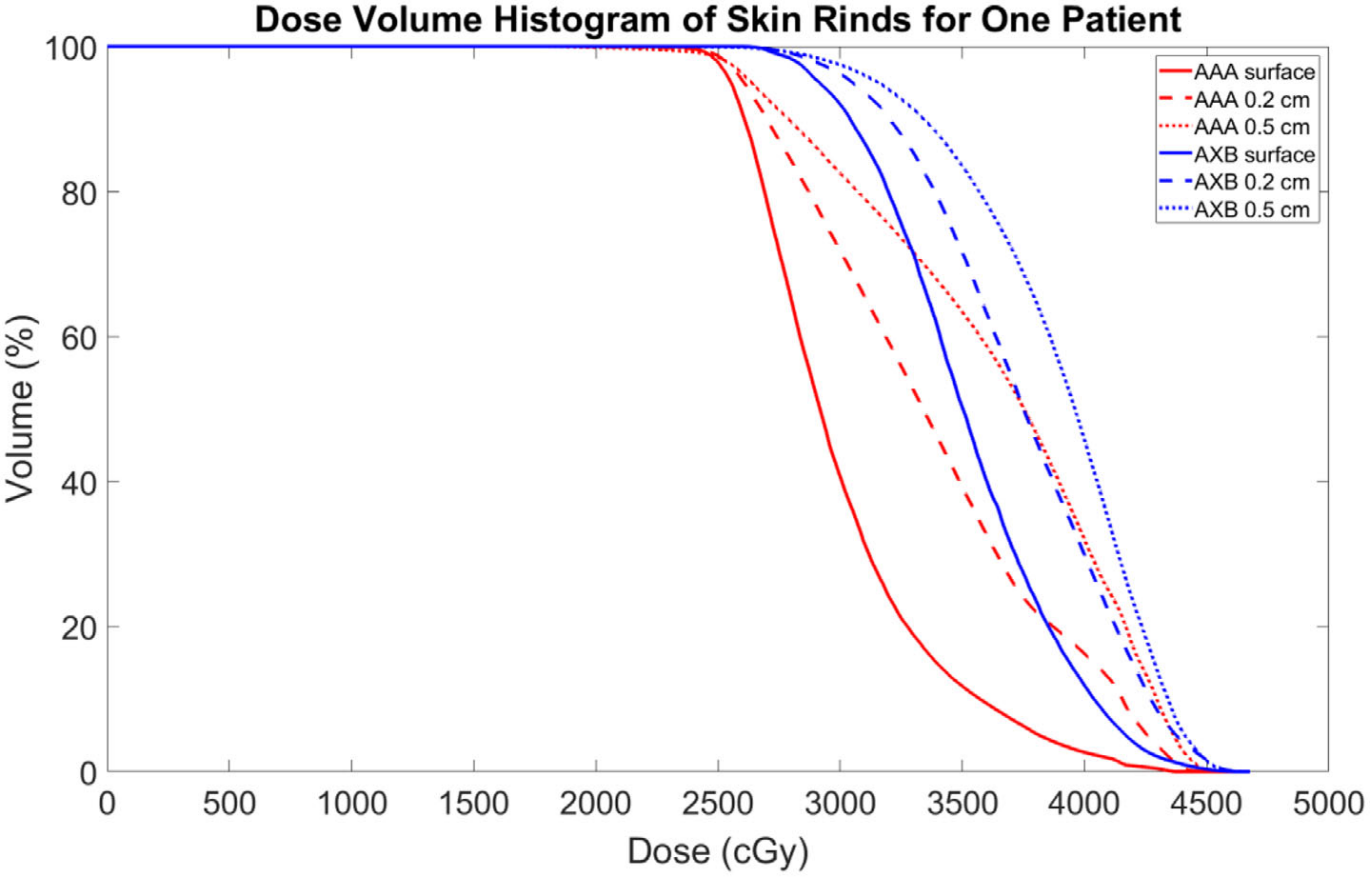
Dose volume histogram of the voxels of the surface layer of the body contour, a 0.2 cm internal skin rind, and a 0.5 cm internal skin rind of the region of contact between the body and the breast cradle for one patient in the study with Analytical Anisotropic Algorithm (AAA) and AcurosXB (AXB).

For AAA and AXB, the DVHs along the surface voxel contour, a 0.2 cm internal skin rind, and a 0.5 cm internal skin rind are shown for one patient in Figure 2. The dose delivered to 50% of the skin structure volume (D50%) is extracted for all 18 patients and summarized in Table 1. For AAA, the mean ± standard deviations of D50% were 79 ± 8% for the surface voxels, 81 ± 6% for the 0.2 cm rind, and 87 ± 4% for the 0.5 cm rind. For AXB, these were 86 ± 4% for the surface voxels, 87 ± 3% for the 0.2 cm rind, and 90 ± 3% for the 0.5 cm rind.

**TABLE 1:** Treatment planning system dose comparison for three skin structures.

	Dose to 50% of the volume (D50%) (percent of prescribed dose)					
	AAA			AXB		
	Surface Voxels	0.2 cm Rind	0.5 cm Rind	Surface	0.2 cm Rind	0.5 cm Rind
Mean ± SD	79 ± 8%	81 ± 6%	87 ± 4%	86 ± 4%	87 ± 3%	90 ± 3%

Dose to 50% of the total volume (D50%) (mean ± standard deviation (SD) across 18 patients, as percent of prescribed dose) of surface layer of body contour, 0.2 cm internal skin rind, and 0.5 cm internal skin rind.

**FIGURE 3:**
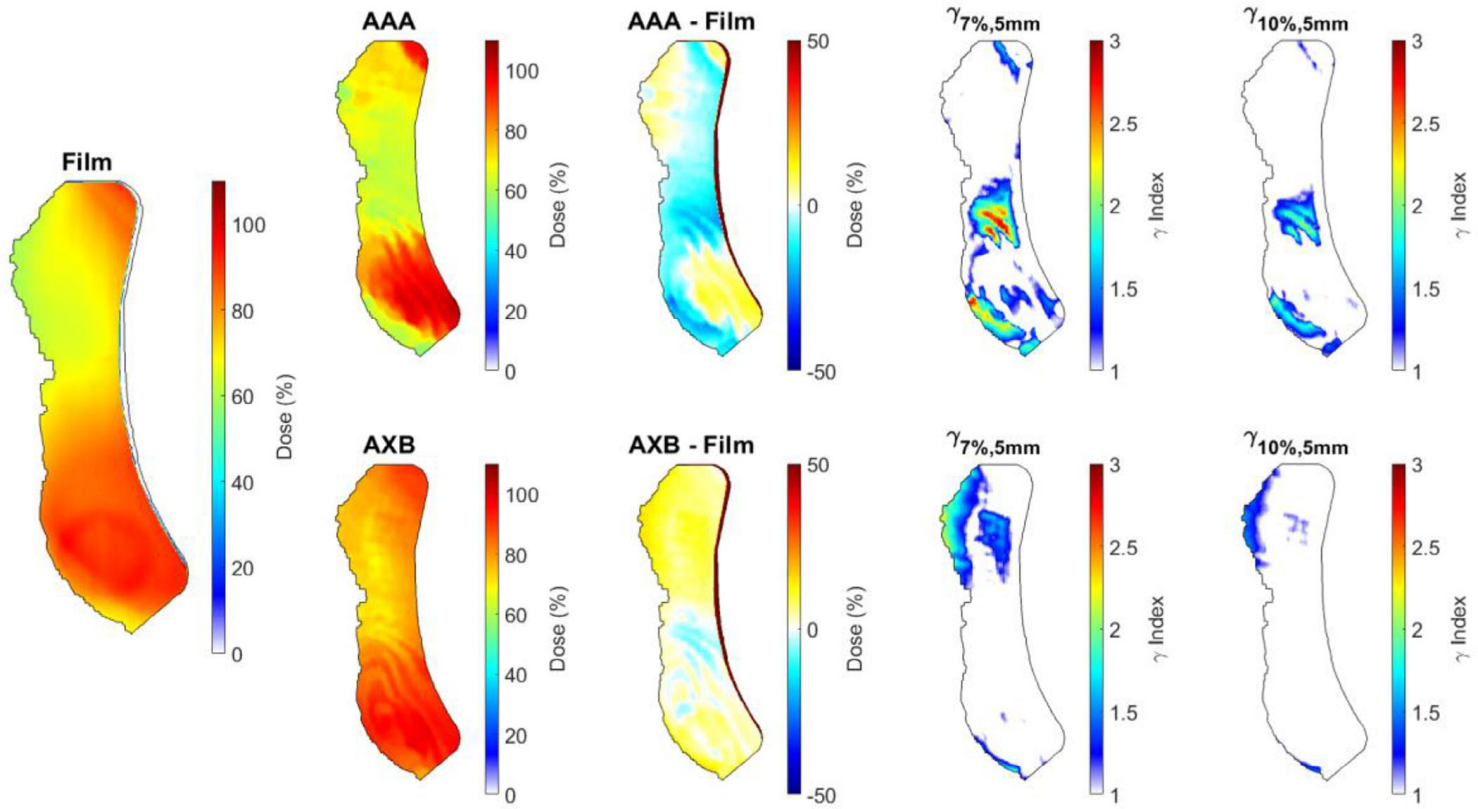
Film, Analytical Anisotropic Algorithm (AAA), and AcurosXB (AXB) dose distributions taken from the 3D breast surface and transformed into 2D. These skin doses are taken from the surface voxel contour of the skin. Black outline shows the region of contact between the breast and the cradle. Dose difference distributions (Treatment Planning System—Film) is shown with a colour scale ranging from a −50% dose difference to +50%. Gamma index analysis (*γ*_7%,5 mm_, *γ*_10%,5 mm_) distributions are shown with colour scale from 1 to 3, with white indicating regions that pass the gamma analysis.

A visual summary of 2D dose distribution results for one patient is shown in Figure 3, with the flattened dose difference maps of calculated and measured dose, and a gamma index analysis. Ridge‐like artefacts appear in the inferior region of the calculated dose distributions extracted from Eclipse for both AAA and AXB. Small regions at the edges of the shape do not overlap, resulting in a steep dose difference near the outline. This is attributed to slight geometric differences in the manually cut film shape and the extracted dose distribution.

**FIGURE 4:**
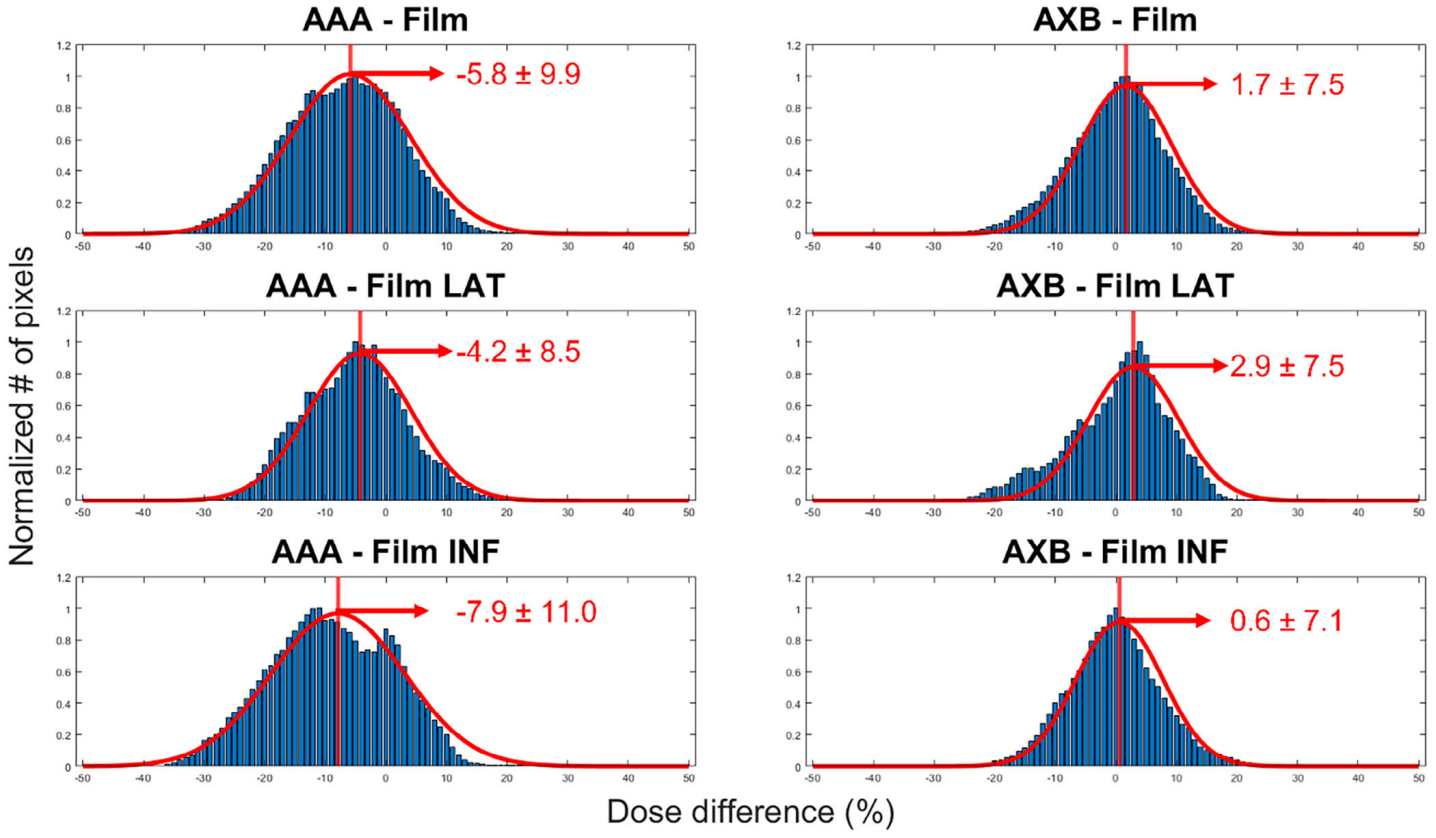
A distribution of dose difference (calculated treatment planning dose—measured film dose) as a percent of prescribed dose for Analytical Anisotropic Algorithm (AAA) and AcurosXB (AXB) over the full, lateral (lat), and inferior (inf) breast regions. The histograms data is generated with bins of 1% dose. Mean ± standard deviation values are quoted in red on figure, with uncertainty of 0.2.

The histogram of dose difference data across all patients is depicted in Figure 4 for AAA versus film and AXB versus film for the full, lateral, and inferior portions of the measured breast. The Gaussian fits yielded *R* ^2^ values greater than 0.96. The Gaussian coefficients [mean ± standard deviation (SD)] had an uncertainty of 0.2%. For AAA versus film, the dose difference mean ± SD were −5.8 ± 9.9% for the full measured surface, −4.3 ± 8.5% for the lateral breast, and −7.9 ± 11.0% for the inferior breast. For AXB versus film, the results were 1.7 ± 7.5% for the full breast, 2.9 ± 7.5% for the lateral breast, 0.6 ± 7.1% for the inferior breast. The *t*‐test yields statistically significant (*p* < 0.05) differences between the AAA versus film and AXB versus film distributions across the full measured breast, the lateral region, and the inferior region.

The impact of the skin rind is demonstrated in Table 2 where the D50% results averaged across the 18 patients are shown. On average, a 0.2 cm skin rind increased the magnitude of the calculated dose at D50% by 2% for AAA and 1% for AXB, and a 0.5 cm skin rind increased the magnitude of the calculated dose at D50% by 8% for AAA and 4% for AXB.

**TABLE 2:** TPS dose versus measured surface dose for AAA and AXB for three skin structures.

	AAA			AXB		
	Surface Voxels	0.2 cm Rind	0.5 cm Rind	Surface	0.2 cm Rind	0.5 cm Rind
TPS dose increase		2%	8%		1%	4%
Calculated (treatment planning system)—Average Measured (film) dose with skin rind shifts						
Full	‐6%	‐4%	2%	2%	3%	6%
Lateral	‐4%	‐2%	4%	3%	4%	7%
Inferior	‐8%	‐6%	0%	1%	2%	5%

Summary of calculated versus measured skin dose as a function of skin rind and TPS algorithm, combining data from Table 1 and Figure 4.

The mean gamma passing rate results for the two dose thresholds across 18 patient cases for both algorithms are shown in Table 3 For AAA versus film, the γ_10%,0.5 cm_ analysis had a passing rate of 73 ± 11% across the full measured region, and for AXB versus film, the γ_7%,0.5 cm_ analysis had a passing rate of 72 ± 14% across the full measured region.

**TABLE 3:** Gamma analysis of film versus treatment planning system dose.

	Gamma passing rate (%)					
	Film vs. AAA			Film vs. AXB		
	Full	Lateral	Inferior	Full	Lateral	Inferior
*γ*_5_ _mm,7%_	61 ± 11%	68 ± 17%	54 ± 15%	72 ± 14%	70 ± 21%	73 ± 15%
*γ*_5_ _mm,10%_	73 ± 11%	80 ± 16%	66 ± 16%	85 ± 12%	83 ± 17%	87 ± 10%

Mean ± SD gamma passing rates for the comparison of Film and AAA and Film and AXB based on the surface voxel contour in the TPS. Gamma index is calculated with distance to agreement of 5 mm and dose tolerances of 7% and 10%.

## 4 DISCUSSION

Skin and surface dose calculations in treatment planning systems have been explored in many studies.^7^, ^18^, ^21^, ^22^, ^23^, ^24^, ^25^ This is the first study to our knowledge to use in vivo dose maps of the breast surface quantitatively registered to the treatment plan to compare measured and calculated dose distributions. The dose maps provide an understanding of how dose calculations may vary across a non‐uniform surface. Patient studies account for different patient shapes and sizes, incident angles, and customized treatment plans, creating a more realistic clinical picture than phantom studies.

While the calculations in this study were conducted with a breast support device, the results can be extended to general epidermal dose at 0.1 cm depth. In our study, a surface pixel analysis was used to study the basal layer. These pixels are sensitive to the partial volume effect, where each surface pixel contains some percentage of air and tissue. An internal skin rind of 0.2–0.5 cm can address this effect by averaging the dose across a thickness within the body contour, reducing the number of pixels in the calculation that are partly filled with air.^18^, ^19^, ^20^ Extending into the dose build up region results in an increased dose estimation as expected, demonstrated in the surface versus 0.2  versus 0.5 cm rind comparison results. When clinically assessing skin toxicity risk or comparing skin dose study results, it could be beneficial to consider these systematic shifts based on the chosen skin rind.

Our results from Figure 4 indicate that in the surface layer of the body contour, AAA underestimates measured dose by 8%, on average, on the inferior breast surface where lateral scatter exits the breast, with better agreement (4% on average) on the lateral breast. AXB is on average within 3% of measured dose on the lateral breast, with better dose agreement (0.6% on average) on the inferior breast surface. With statistically significant differences in accuracy of dose calculation, it is important to note that AXB is distinctly closer to the film skin measurements than AAA in the surface layer of pixels. Both AAA and AXB unfolded dose maps show periodical ridge‐like artefacts in the inferior breast region. These are likely attributed to the 12° angle of the breast cradle. CT slices are vertical, so the plane of the cradle passes through a number of 0.5 cm thick CT slices in the superior‐inferior direction. It is likely these artefacts are being introduced by the TPS in the reconstruction process. These artefacts increase the variation in dose differences shown in Figure 4. The periodic artefacts informed the choice of a distance to agreement of 0.5 cm for the gamma index criteria. The gamma index analysis results with dose thresholds of 7% and 10% were consistent with the standard deviations of the mean of the dose difference analysis, confirming the correct registration of our 2D images.

Cao and colleagues measured surface dose with film on a cubic phantom, comparing it against Eclipse calculations with a 0.1 cm calculation grid at incident beam angles of 0°, 30°, 60°.^21^ Compared to film, AAA overestimated dose at the superficial surface by 3% at 0°, and underestimated dose by 2% for 30° and by 13% at 60°. AXB overestimated dose by 1% at 0°, 3% at 30°, and 3% at 60°. This study demonstrated that AAA dose calculation is dependent on beam angle, with calculations varying from underestimating to overestimating the measured dose as the incident angle increased. In realistic tangential beam treatments, patients have curved breast geometry and the divergent radiation beam hits the skin's surface at a range of angles. The average agreement between measured epidermis dose and AAA calculations from our study fall within this range of under/overestimation depending on beam incidence. Lim and colleagues created a breast shaped phantom with a 0.5 cm skin rind, measuring surface dose with optically stimulated luminescence dosimeters (OSLD) that are contoured in Eclipse.^24^ The study showed that AAA overestimates OSLD surface measurements by 3.9% and AXB by 2.5%. The results from this study are consistent with our analysis with a 0.5 cm skin rind for AAA, and within clinical tolerance for AXB. These phantom studies are a valuable starting point in understanding skin dose calculations.

In a previous study, our group proposed skin dose constraints to reduce the incidence of moist desquamation in breast radiotherapy.^15^ This study demonstrated a separation of 10% in prescribed dose in DVH curves of patients who developed moist desquamation and those that did not. To implement the dose constraints developed in this study, a TPS agreement with film of ± 5% would be required. AAA can reproduce film with an accuracy of 5% if a 0.5 cm rind is used inside the skin contour, otherwise it underestimates the dose, particularly in the inferior breast region. AXB matches film within an accuracy of 5% using either the external skin contour alone or a 0.2 cm rind, but a 0.5 cm rind will result in an overestimation of epidermal dose.

In this study, the lateral breast is the beam entrance surface. The dose in the inferior breast surface is largely due to lateral scatter. AAA and AXB calculate these doses in different ways. The results of this study indicate that AAA's dose scaling along the beams forward trajectory at the entrance air‐tissue interface performs better than the lateral scaling responsible for dose at the tissue‐air interface on the inferior breast. AXB's direct solution to LBTE results in more accurate dose calculations along the entirety of the measured skin surface. AAA scales dose for inhomogeneous media based on the different electron densities but does not account for atomic number. This approximation works well enough in general for AAA to be a widely used algorithm. However, the approximation impacts dose deposition and particle transportation, and the results of this study indicate that in the instance of the tissue‐air interface, epidermal dose calculations are more accurate with AXB's methods of directly solving the LBTE.

## 5 CONCLUSION

AAA is often used clinically in planning standard breast treatments. Based on the results from this study, it is recommended that AAA epidermal dose is calculated with a 0.5 cm skin rind for clinically acceptable epidermal dose calculations. A rind ≤ 0.2 cm is recommended for clinically acceptable calculation of the epidermal dose using AXB. These results should be considered during treatment planning, especially for patients who are at a high risk of developing skin reactions.

## AUTHOR CONTRIBUTIONS

Aria Malhotra acquired TPS data, performed study analysis and wrote the manuscript in consultation with Emilie E. Carpentier. Emilie E. Carpentier acquired and analyzed film data. Cheryl Duzenli designed study, and supervised the analysis and manuscript writing.

## CONFLICT OF INTEREST STATEMENT

United States patent No. 16/487694: Apparatus for positioning a breast for radiation treatment
